# Healthcare students’ hand hygiene knowledge, beliefs and practices

**DOI:** 10.1186/1753-6561-5-S6-P113

**Published:** 2011-06-29

**Authors:** TF Van De Mortel, E Apostolopoulou, G Petrikkos, E Hedberg, B Edlund, H Wijk

**Affiliations:** 1 Healtha and Human Sciences, Southern Cross University, Lismore, Australia; 2 Department of Nursing, University of Athens, Athens, Greece; 3 Department of Internal Medicine, University of Athens, Athens, Greece; 4 Department of Nursing, Health & Culture, University West, Trollhattan, Sweden; 5 Department of Public Health & Caring Sciences, Uppsala University, Uppsala, Sweden; 6 Sahlgrenska Academy, Gothenburg University, Gothenburg, Sweden

## Introduction / objectives

Hand hygiene (HH) compliance reduces the incidence of nosocomial infection, however, little research has been conducted on factors that influence healthcare students’ HH practices. The study aims were to examine the relationships between healthcare students' HH knowledge, beliefs and practices, the ways they were educated and assessed on HH, and their perceptions of the importance given to HH in the curriculum.

## Methods

A HH questionnaire was administered to 1485 nursing and medical students from 19 universities in Australia, Sweden and Greece. The General Linear Model was used to examine the relationships between study variables.

## Results

Knowledge scores were significantly influenced by the frequency of HH assessment and the number of methods used to teach HH (F=3.2;p = .04). HH practices were significantly influenced by HH beliefs, knowledge, assessment frequency, number of teaching methods, perceptions of the importance of HH as an infection control measure and the importance given to HH in the curriculum (F=84.7;p < .01), although HH beliefs were the greatest predictors of practice. Hand hygiene beliefs were significantly influenced by HH knowledge, by students’ perceptions of the importance given to HH in the curriculum and by supervisors and facilities, and the importance of HH as an infection control measure (F=46.3;p < .01).

## Conclusion

While repeated HH education and assessment offers a means of improving undergraduate health care students' HH knowledge and practices, students' beliefs about HH had a greater impact on their HH practice than their HH knowledge did. Encouraging more positive HH beliefs may be a more effective way of improving HH practice.

## Disclosure of interest

None declared.

